# Short-term prediction of PM2.5 concentration by hybrid neural network based on sequence decomposition

**DOI:** 10.1371/journal.pone.0299603

**Published:** 2024-05-10

**Authors:** Xiaoxuan Wu, Jun Zhu, Qiang Wen

**Affiliations:** 1 School of Artificial Intelligence and Big Data, Economic and Technological Development Zone, Hefei University, Hefei City, Anhui, China; 2 Key Laboratory of Intelligent Building and Building Energy Efficiency, Anhui Jianzhu University, Hefei, China; 3 School of Computer and Information, Hefei University of Technology, Hefei, China; Kanazawa University, JAPAN

## Abstract

Accurate forecasting of PM2.5 concentrations serves as a critical tool for mitigating air pollution. This study introduces a novel hybrid prediction model, termed MIC-CEEMDAN-CNN-BiGRU, for short-term forecasting of PM2.5 concentrations using a 24-hour historical data window. Utilizing the Maximal Information Coefficient (MIC) for feature selection, the model integrates Complete Ensemble Empirical Mode Decomposition with Adaptive Noise (CEEMDAN), Convolutional Neural Network (CNN), and Bidirectional Recurrent Gated Neural Network (BiGRU) to optimize predictive accuracy. We used 2016 PM2.5 monitoring data from Beijing, China as the empirical basis of this study and compared the model with several deep learning frameworks. RNN, LSTM, GRU, and other hybrid models based on GRU, respectively. The experimental results show that the prediction results of the hybrid model proposed in this question are more accurate than those of other models, and the R2 of the hybrid model proposed in this paper improves the R2 by nearly 5 percentage points compared with that of the single model; reduces the MAE by nearly 5 percentage points; and reduces the RMSE by nearly 11 percentage points. The results show that the hybrid prediction model proposed in this study is more accurate than other models in predicting PM2.5.

## Introduction

Despite the rapid advancements in global science and technology, the worldwide environment faces degradation, with air pollution emerging as a prominent concern. PM2.5, a notable air pollutant, originates from both natural sources—such as aeolian dust, volcanic eruptions, forest fires, sea salt, pollen, and microbial activities—and anthropogenic activities. Notably, human-induced emissions remain the predominant contributor to PM2.5 levels. These emissions also release gaseous pollutants, which can transform into PM2.5 via complex chemical reactions. Consequently, rising PM2.5 concentrations degrade air quality annually. This degradation not only diminishes human visual acuity, resulting in challenges like vehicular accidents, but prolonged exposure to elevated PM2.5 levels also jeopardizes respiratory and cardiovascular health [1, 2]. Thus, the accurate prediction of PM2.5 concentrations is pivotal for effective air pollution mitigation and public health advisories, enabling individuals to make informed travel decisions, minimize PM2.5-related health risks, and safeguard against extreme weather events [3, 4].

Predicting PM2.5 concentrations is fundamentally a time-series forecasting challenge, wherein historical and current data inform future PM2.5 levels [5]. The intricacy of such predictions arises from the PM2.5 data being nonlinear and nonstationary due to various external influencers [6–8]. Research methodologies span traditional time series modeling, machine learning, and deep learning.

Earlier studies favored time series-centric models like the autoregressive sliding average (ARMA) and the autoregressive moving average differential equation (ARIMA) for forecasting PM2.5 concentrations [9]. However, these models often falter in handling non-linear target series, leading to unsatisfactory predictions. Shallow machine-learning networks, such as artificial neural networks, grey neural network models, and radial basis function neural networks, have shown promise in modeling complex non-linear time-series relationships. Yet, their simplistic structures introduce issues like local minima entrapment for BP neural networks and gradient challenges for recurrent neural networks [10]. Deep learning, meanwhile, has exhibited prowess in managing the nonlinear complexities inherent in such time series. Luo et al. [11] employed both deep learning and machine learning in an image-based approach to enhance the detection of PM2.5. They developed an end-to-end model combining a convolutional neural network with a gradient boosting machine. Meanwhile, Li et al. [12] introduced the AC-LSTM model, a fusion of one-dimensional Convolutional Neural Network (CNN), Long Short-Term Memory Network (LSTM), and Attention-Based Network. This model incorporates air pollutant concentrations, meteorological data, and PM2.5 levels from nearby monitoring stations as input. It harnesses the CNN-LSTM network to assimilate multivariate time series data on air quality, focusing on their spatiotemporal interrelations. The attention mechanism gauges the impact of past states on future PM2.5 concentrations, refining predictive precision through an attention-based hierarchy. In another study, Li et al. [13] introduced a gated cell model integrated with reinforcement learning, specifically the SAE-GRU method. Here, the sparse autoencoders(SAE) distils low-dimensional features of PM2.5 data, while the gated recurrent units(GRU) refines subseries prediction. Zhao et al. [14], on the other hand, presented a spatiotemporal air quality prediction model that leverages abundant environmental data and an LSTM neural network for future air quality forecasts.

While deep learning networks possess the theoretical capability to model complex, non-linear time-series data, their performance is constrained by limitations such as training data volume, network size, and the stochastic and non-smooth nature of PM2.5 concentration series. To address this, the data is preprocessed through empirical mode decomposition (EMD), which decomposes the irregular time-series into multiple intrinsic modal functions (IMFs) and a residual component (RES). Notably, EMD obviates the need for a priori basis functions, instead employing an a posteriori approach that adapts to changing data characteristics [15]. Despite its advantages, EMD suffers from modal aliasing. To mitigate this, our study employs complete ensemble empirical mode decomposition with adaptive noise (CEEMDAN).

In atmospheric science, PM2.5 emanates from both primary and secondary sources, with the latter including chemical reactions that yield PM2.5 as a byproduct. Consequently, gases such as NO2, SO2, and O3 play a significant role in PM2.5 concentration [16]. Furthermore, PM2.5 levels are influenced by local climatic conditions [17] as well as air quality and meteorological factors [18, 19]. As such, optimizing prediction models may necessitate feature selection focused on variables with a high degree of correlation to PM2.5 levels, thereby mitigating the impact of irrelevant variables [20, 21].

**Fig 1:**
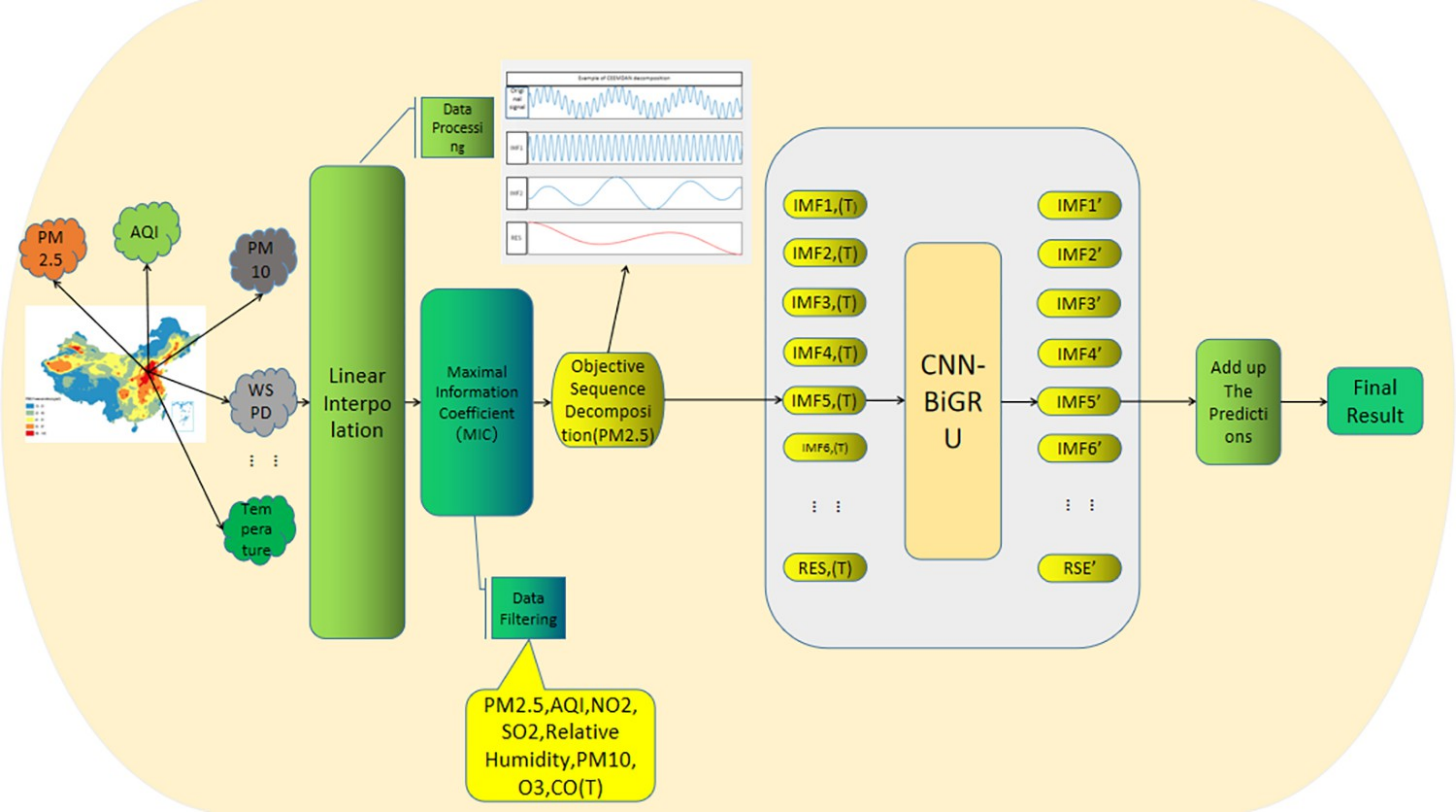
Hybrid model structure diagram.

In the hybrid model presented herein, maximal information coefficients (MICs) are employed to identify features most correlated with PM2.5. These features and historical PM2.5 time-series data, which are inherently non-stationary, are decomposed into eigenmode functions and a residual via CEEMDAN. These processed elements are integrated into a hybrid neural network alongside filtered feature data, facilitating model training and prediction. Subsequently, the individual predictions are aggregated for short-term PM2.5 concentration forecasting. The model architecture is delineated in Fig 1.

The key contributions of this study are as follows:

1. The use of MIC allows for capturing non-linear correlations between PM2.5 concentrations and both meteorological and atmospheric factors, enhancing predictive accuracy.
2. CEEMDAN-based decomposition circumvents the need for parameter tuning in wavelet analysis and alleviates modal aliasing seen in traditional EMD methods, yielding a more accurate data representation.
3. The hybrid model amalgamates the strengths of various algorithms, honing the precision of the predictions.

## Data processing

The experimental dataset utilized in this study was obtained from the environmental cloud of Nanjing Yunchuang Big Data Technology Co., LTD. We accessed hourly meteorological records (comprising weather conditions, air temperature, felt temperature, air pressure, humidity, rainfall, wind direction, and wind speed) and hourly air quality monitoring data (PM10, CO, SO2, NOx, O3) for Beijing, spanning from January 1, 2016 to December 31, 2016. The air quality monitoring data refers to the hourly data from 12 monitoring locations, resulting in a total of 8,784 records for each monitoring point, data from one of these monitoring sites was selected for this study though some data are missing due to uncontrollable factors. Missing data is mainly missing meteorological data, including missing meteorological data on a certain day and hour or missing wind direction data on a certain day and hour. If the entire meteorological data of a certain hour on a certain day is missing, the data is deleted. If the wind direction data of a certain hour on a certain day is missing, the linear interpolation method is used to complete the data. The mathematical principle governing this approach is described in Eq (1), wherein the missing value at a specific time (x) is substituted by its corresponding value at point F(x). The dataset is a time-series with 13 features related to air quality and meteorological factors, totaling 8784 instances at hourly intervals. For our experiments, we partitioned the data into two sets: 70% for training and 30% for testing.


$$F_{\left(x\right)}=ax+b$$ (1)

## Methodology

### Feature selection

Air quality and meteorological parameters critically influence the dispersion of air pollutants. Rigorous assessment of these parameters is fundamental for accurate PM2.5 concentration forecasting. In our current investigation, we employed the MIC to ascertain relationships between individual pollutant attributes and PM2.5 levels. The MIC represents an innovative technique for identifying nonlinear correlations amidst variables, characterized by its expansive applicability, computational efficiency, and remarkable robustness. Fundamentally, MIC discretizes the interplay of two variables within a bidimensional framework visualized via a scatter plot. This space is segmented into defined intervals across the X and Y axes, situating scatter points within their respective grid cells. This method effectively addresses the mutual information joint likelihood challenges. MIC values oscillate between 0 and 1, with ascending values signifying intensified correlations.

The computation of the MIC proceeds as follows:

(1) For given variables i and j, a scatterplot grid of X and Y in column a and row b is established to calculate the mutual information (MI). The MI between two variables x and y is defined as per Eq (2), where p(x,y) represents the joint probability distribution of x and y.

$$MI(x;y)=\int p(x,y)\log_{2}\frac{p(x,y)}{p(x)p(y)}dxdy$$ (2)

(2) Subsequent to the MI calculation, a normalization process is conducted.
(3) The MIC value is then determined by selecting the maximum MI value across different scales.

$$mic(x;y)=\underset{a*b<B}{\max}\frac{MI(x;y)}{\min(a,b)}$$ (3)

In the above, a and b represent the number of divisions in the x and y directions of the lattice, respectively. B is a variable whose magnitude approximates the 0.6 power of the data volume.

**Fig 2:**
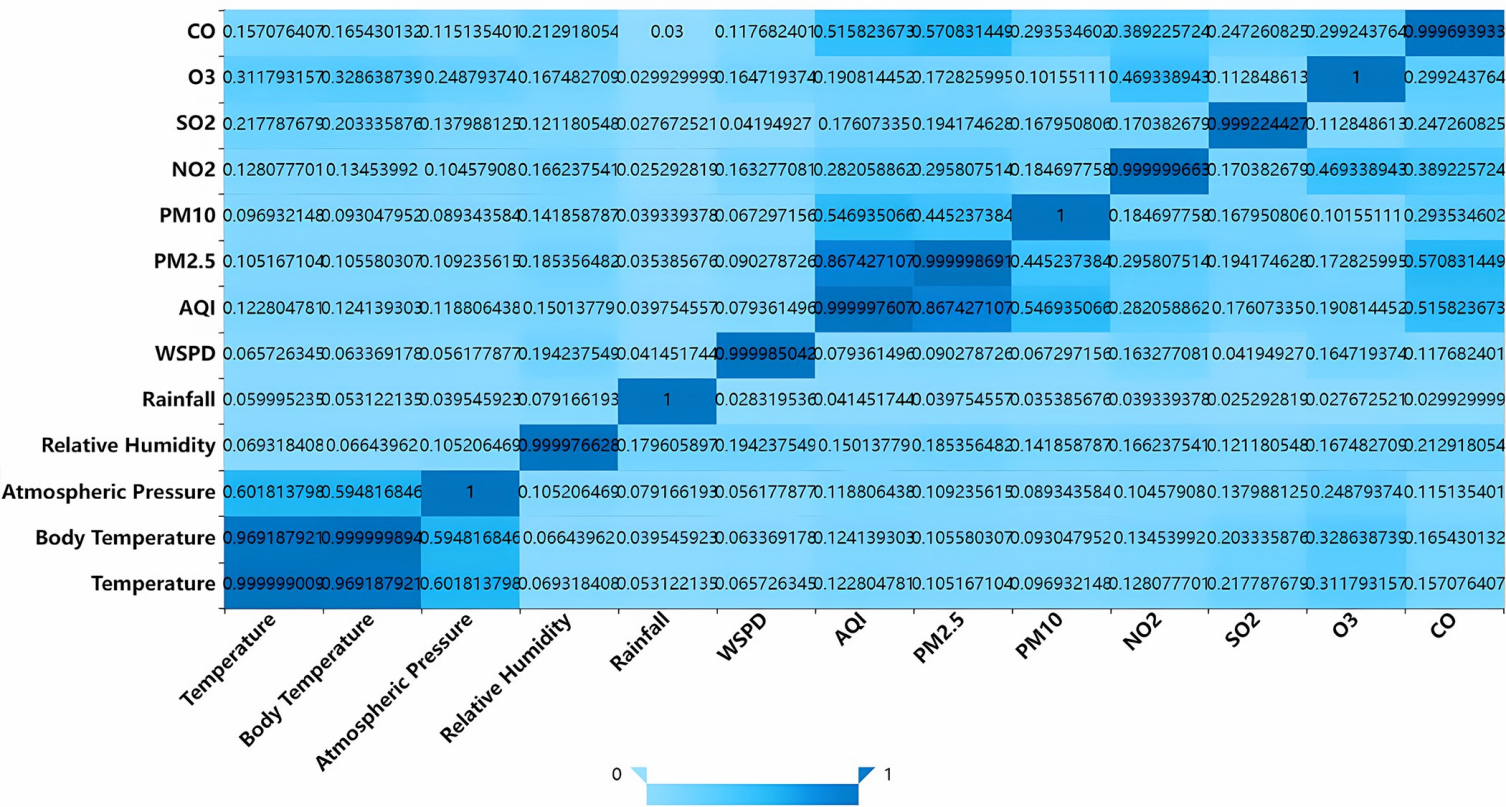
Correlation heat map.

**Fig 3:**
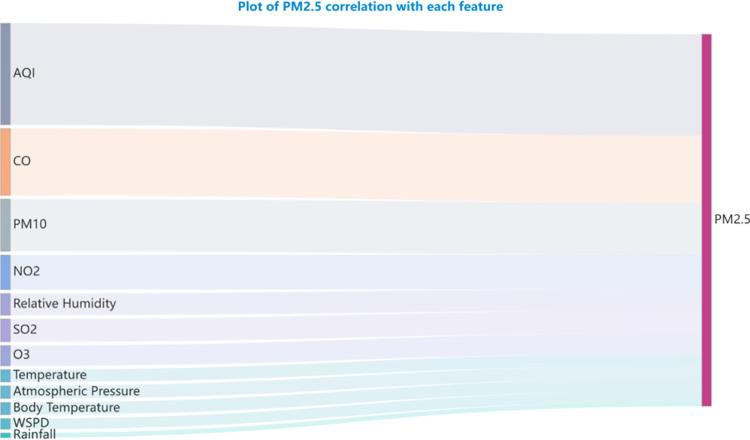
Feature correlation diagram.

Fig 2 elucidates the interrelationships between thirteen salient air quality and meteorological parameters. Further, Fig 3 quantifies the association magnitude of individual features with PM2.5. Notably, AQI, CO, and PM10 manifest the most pronounced affiliations with PM2.5, while parameters like air temperature, body temperature, barometric pressure, and rainfall exhibit marginal influence, underscoring their diminished role in modulating PM2.5 variations. The data foundation of our analysis stems from Beijing’s meteorological records for the entirety of 2016. Given Beijing’s geographical position in northern China, which is typically marked by limited precipitation, one can deduce that the influence of rainfall on PM2.5 concentrations is comparatively negligible. This observation underscores the pertinence of the feature set chosen via the MIC method.

**Fig 4:**
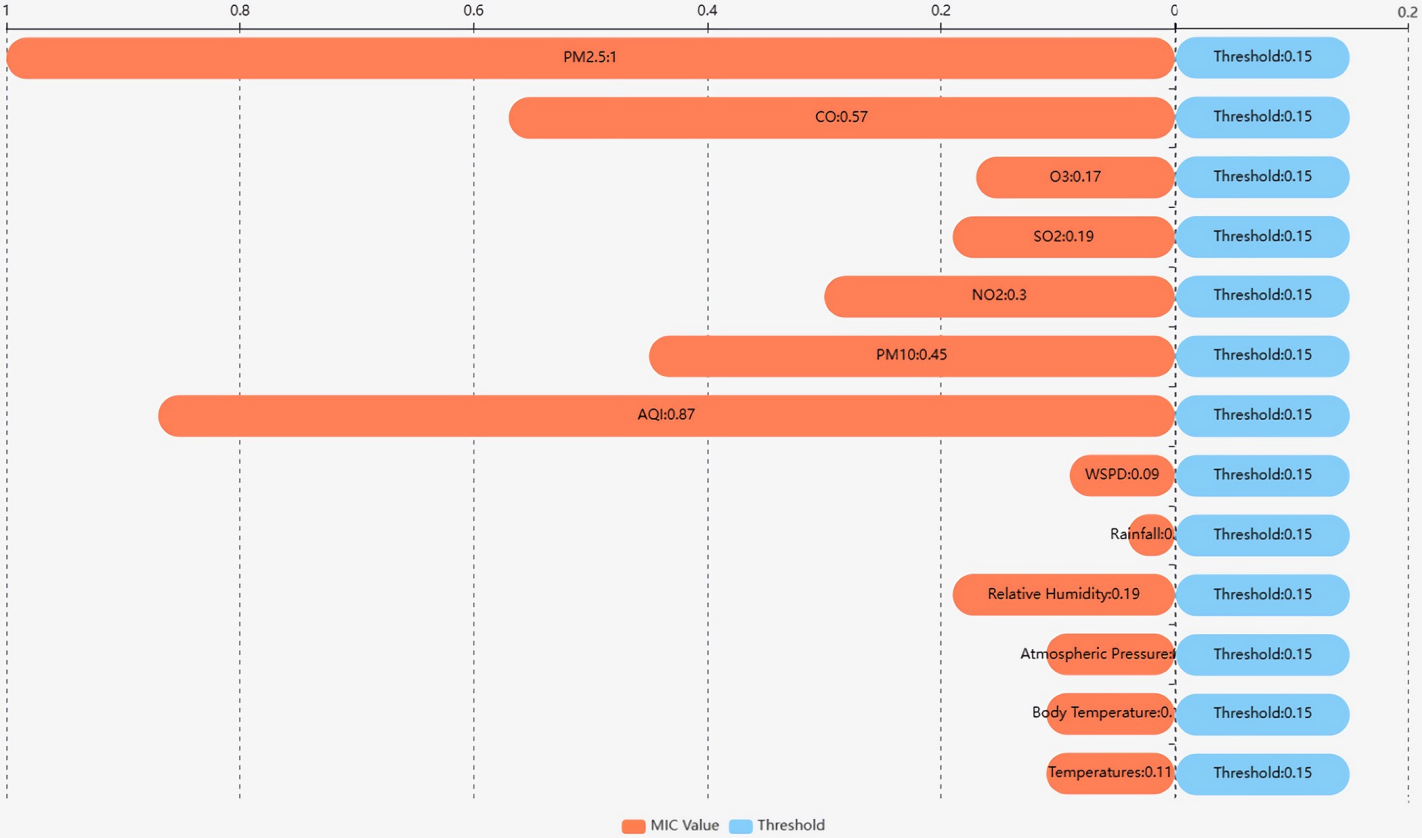
Feature threshold comparison chart.

A threshold of 0.15 was established, informed by the geographical context of the dataset and the median income indicator for each feature. By juxtaposing MIC values against this threshold, as illustrated in Fig 4, eight salient features emerged: PM2.5, AQI, CO, PM10, SO_2_, O_3_, NO_2_ and relative humidity, all of which are detailed in Table 1.

**Table 1:** MIC values for each feature and PM2.5 concentration.

Features	MIC Value
Relative Humidity	0.19
AQI	0.87
PM10	0.45
NO2	0.30
SO2	0.19
O3	0.17
CO	0.57
PM2.5	1.00

### Data decomposition

**Fig 5:**
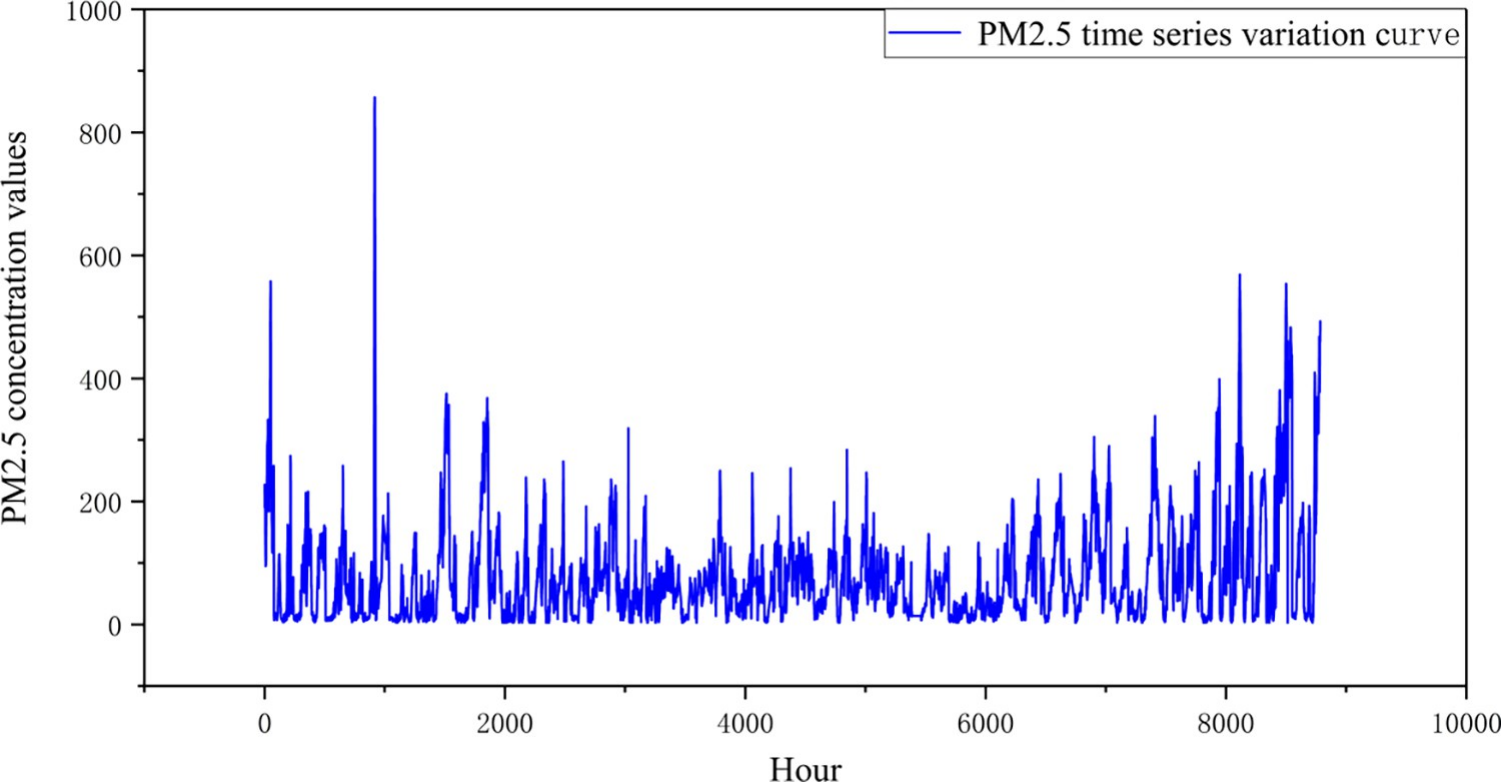
Time series plot of PM2.5 concentration.

The time series data manifests distinctive attributes such as long-term trends, cyclical patterns, and seasonal shifts, culminating in pronounced data volatility. An example of this can be seen in Fig 5, depicting the hourly PM2.5 concentrations spanning 1 January to 31 December 2016 in Beijing. Notably, this particular year was selected for investigation. A visual analysis of Fig 5 highlights that the waveform distribution is heterogeneous, with the series showcasing substantial variances and a lack of continuity.

Historically, strategies for decomposing time series have been formulated to distill multi-dimensional insights and ascertain the ramifications of diverse factors on the series. In light of this, our study leverages the CEEMDAN technique, CEEMDAN can avoid modal mixing conditions during the decomposition process, tailored for parsing non-stationary time series into multiple stationary counterparts. Mode mixing refers to the phenomenon where different intrinsic mode functions (IMFs) extracted by EMD contain mixed information from different scales and frequencies, leading to inaccurate decomposition results. In EEMD, mode mixing is a common issue that can affect the accuracy of the decomposition. CEEMDAN addresses this problem by introducing an adaptive noise component that helps to reduce mode mixing and improve the accuracy of the decomposition. This adaptive noise component is added to the original signal to create an ensemble of signals, which are then decomposed using EMD. The adaptive noise component helps to separate the mixed information and improve the decomposition results by reducing mode mixing. CEEMDAN differs from EEMD in terms of mode mixing by including an adaptive noise component to reduce mode mixing and improve the accuracy of the decomposition.

The detailed principles and sequential steps of the decomposition process are outlined below:

(1) Gaussian white noise is incorporated into the signal targeted for decomposition, y(t), generating a novel signal, y(t)+(−1)^q^*ε*v^j^(t), where q = 1,2. Subsequently, EMD decomposition of this newly formed signal, inclusive of Gaussian white noise, is performed to yield the first-order intrinsic modal component, C_1_.

$$E(y(t)+{\left(-1\right)}^{q}\varepsilon v^{j}(t))=C_{1}^{j}(t)+r^{j}$$ (4)

(2) The first eigenmode component of the CEEMDAN decomposition is ascertained by averaging the N modal components derived from the EMD decomposition.

$$\bar{C_{1}(t)}=\frac{1}{N}\sum_{j=1}^{N}{C_{1}}^{j}(t)$$ (5)

(3) The original signal’s residue is obtained by subtracting the first eigenmode function.

$$r_{1}(t)=y(t)-\bar{C_{1}(t)}$$ (6)

(4) Positive and negative paired Gaussian white noise is integrated into r_1_(t) to generate a new signal. This signal then serves as a substrate for a subsequent round of EMD decomposition, resulting in the first-order modal component, D_1_. Consequently, the second eigenmode component of the CEEMDAN decomposition is derived.

$$\bar{C_{2}(t)}=\frac{1}{N}\sum_{j=1}^{N}{D_{1}}^{j}(t)$$ (7)

(5) The residuals from the initial stage are subtracted from the second modal component.

$$r_{2}(t)=r_{1}(t)-\bar{C_{2}(t)}$$ (8)

(6) The aforementioned steps are iteratively repeated until the residual signal manifests as a monotonic function, at which point further decomposition is infeasible, signaling the termination of the algorithm. At this juncture, the total count of intrinsic modal components procured is denoted as K. The original signal, y(t), is then decomposed as delineated in Eq 9.

$$y(t)=\sum_{k=1}^{K}\bar{C_{k}(t)}+r_{k}(t)$$ (9)

In this equation, E_i_(•) represents the i-th eigenmode component post-EMD decomposition, while the i-th eigenmode component following CEEMDAN decomposition is denoted as $\bar{C_{i}(t)}$. V^j^ signifies the j-th Gaussian white noise signal, conforming to a standard normal distribution, where j = 1,2,3,⋯,N represents the instances of white noise incorporation. *ε* symbolizes the Gaussian noise weight coefficient, and y(t) signifies the signal targeted for decomposition.

**Fig 6:**
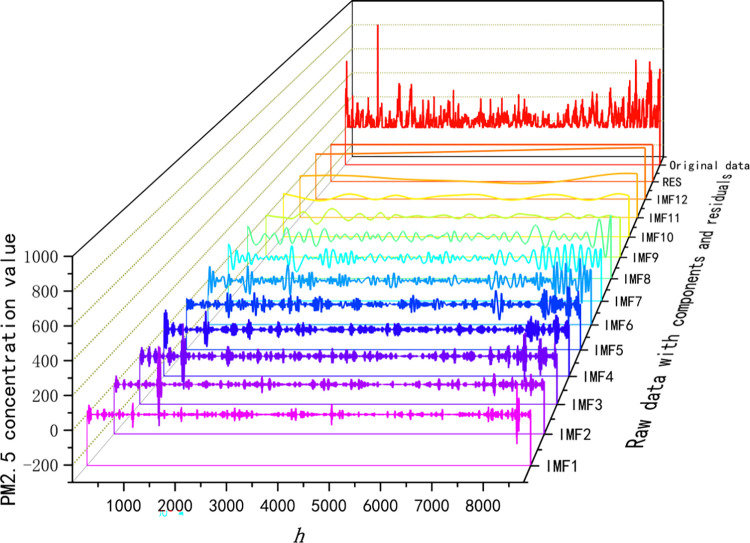
Sequential decomposition of PM2.5 concentrations.

In the course of time series refinement, the PM2.5 concentration time series undergoes decomposition via the CEEMDAN algorithm. This decomposition yields 12 IMFs and a single residual, as illustrated in Fig 6. Each IMF encapsulates a smooth time series of PM2.5 concentration across distinct frequency domains, with no overlap occurring between any two IMFs. The IMFs decomposed by CEEMDAN represent the inherent patterns or features of the original signal on different time scales. Each IMF represents an oscillation or change in the original signal on a particular time scale. These IMFs can be interpreted as different frequency components in the data, thus providing information about the intrinsic structure and change patterns of the data. In CEEMDAN, due to the introduction of an adaptive noise component, the decomposed IMFs are more robust and better able to capture the true patterns in the data compared to conventional EMD. Therefore, the IMF decomposed by CEEMDAN can more accurately reflect the oscillation characteristics and change patterns of the data. The original series can be reconstituted by aggregating all the IMFs along with the final residual component.

### CNN model

**Fig 7:**
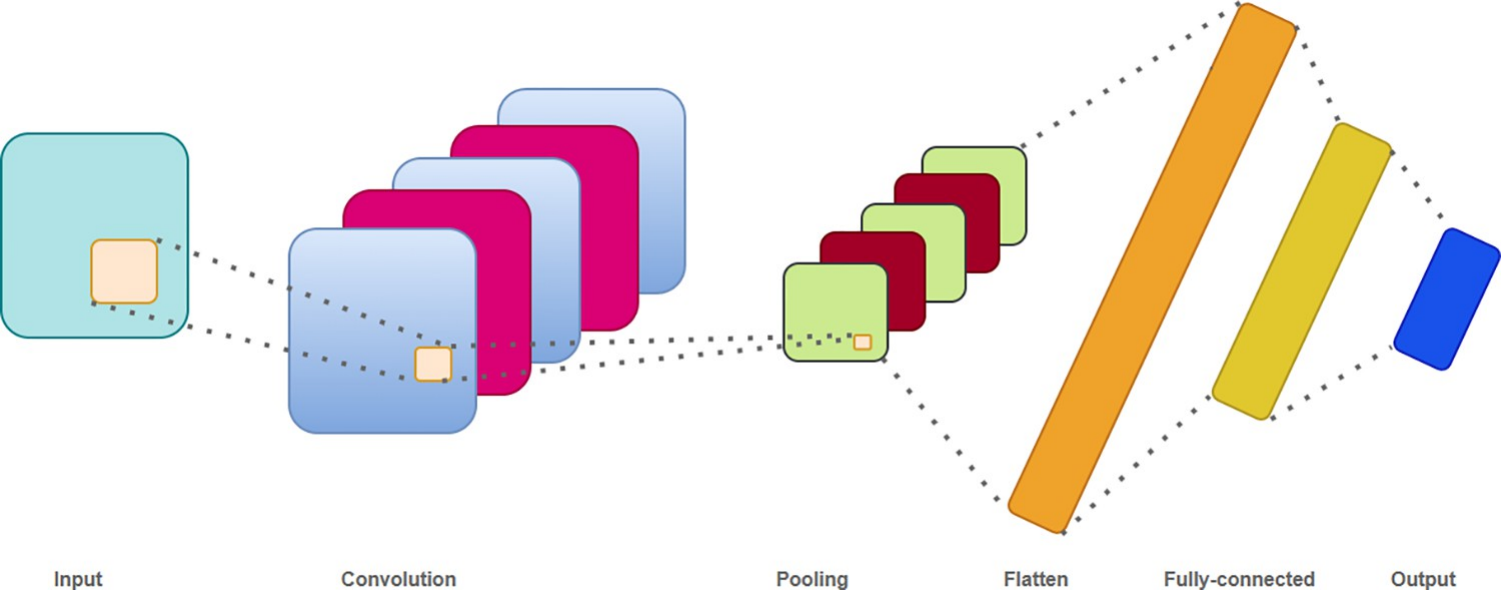
CNN model diagram.

Numerous neural networks have proven successful in the realm of deep learning, among which CNNs are particularly noteworthy due to their capacity for feature extraction and weight sharing. CNNs can be classified into three lattice structures: one-dimensional, two-dimensional, and three-dimensional CNNs, each serving unique functions. One-dimensional CNNs are primarily harnessed for sequence data processing, making them apt for the methodology adopted in this study. Conversely, two-dimensional CNNs are typically employed for image recognition tasks, while three-dimensional CNNs are predominantly used for video recognition. Thus, in this study, we leverage one-dimensional CNNs. These networks exhibit exceptional efficacy in text time series analysis, as they can adjust the size and orientation of the convolutional kernel based on the data and text dimensions, enabling a deep exploration of the samples. The primary process of a one-dimensional CNN is depicted in Fig 7.

The fundamental components of the CNN comprise the convolutional layer, pooling layer, and fully connected layer. Within the convolutional layer, a convolutional kernel is used to execute convolutional operations on the processed data, extracting features and generating the corresponding feature maps. The pooling layer serves to reduce the model’s complexity, enabling simpler and quicker computations, while preserving essential data features. The fully connected layer maps the preceding layers to their respective positions, enhancing the overall integrity of the model’s structure.

### GRU model

**Fig 8:**
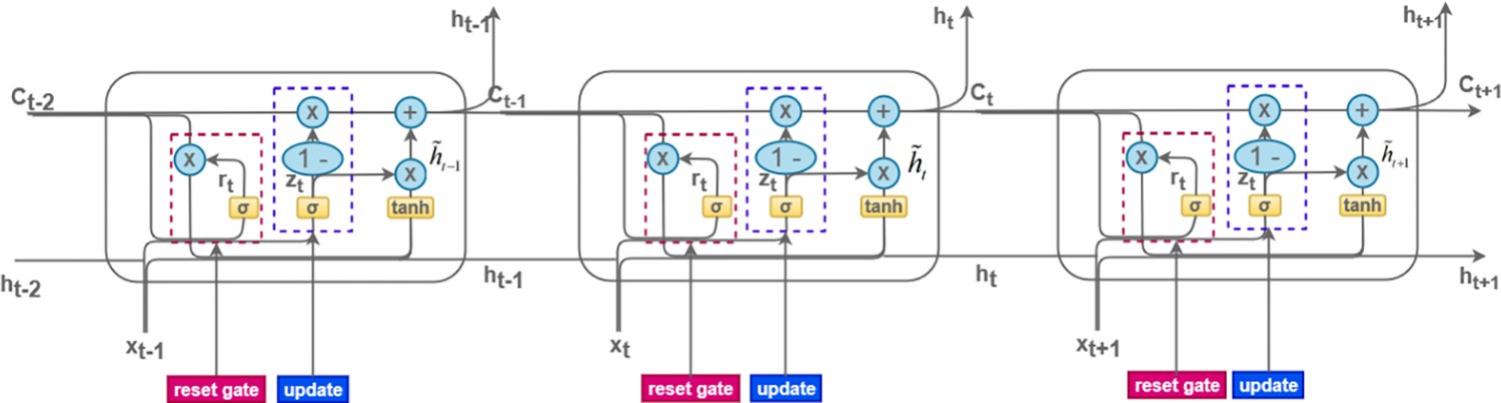
GRU network structure diagram.

In the data processing stage, Recurrent Neural Networks (RNN) may face the challenge of gradient explosion due to the large amount of data and the long duration of the sequence [22, 23]. In 1997, Horchreiter and Schmidhuber proposed a special type of Recurrent Neural Network (RNN) called LSTM. The network is a modified structure of simple RNN and solves the problem of gradient explosion and gradient vanishing that occurs in the traditional RNN.LSTM network processing series uses three basic gates: input gate forget gate and output gate. LSTM has the ability to learn long-term dependencies from the input sequence data. The LSTM network uses three basic gates: input gate, forget gate, and output gate. LSTM has the ability to learn long-term dependencies from input sequence data [24, 25]. Compared with LSTM, GRU usually has fewer parameters, so it can speed up the training time and reduce the computational complexity; GRU’s performance in capturing dependencies in long sequences in a short period of time is not comparable to that of LSTM, and GRU is usually easier to train than LSTM, especially when dealing with smaller datasets or limited computational resources. Combining these factors, we choose GRU as our prediction model. Fig 8 shows the schematic diagram of GRU.

The GRU model is characterized by two gates: the update gate and the reset gate. The reset gate integrates the new input with prior memory, as detailed in Eq (10):

$$r_{t}=\sigma (W_{r}\cdot [h_{t-1},x_{t}])$$ (10)

The update gate modulates the influence of the information from the preceding time step on the current step, effectively dictating the retention and propagation of past data. This gate primarily updates the model’s memory, as formulated in Eq (11):

$$z_{t}=\sigma (W_{r}\cdot [h_{t-1},x_{t}])$$ (11)

Here, σ signifies the activation function, while W_T_ and W_Z_ represent the weight matrix. h_t−1_ denotes the preceding state, and x_t_ signifies the current input value.

### BiGRU model

**Fig 9:**
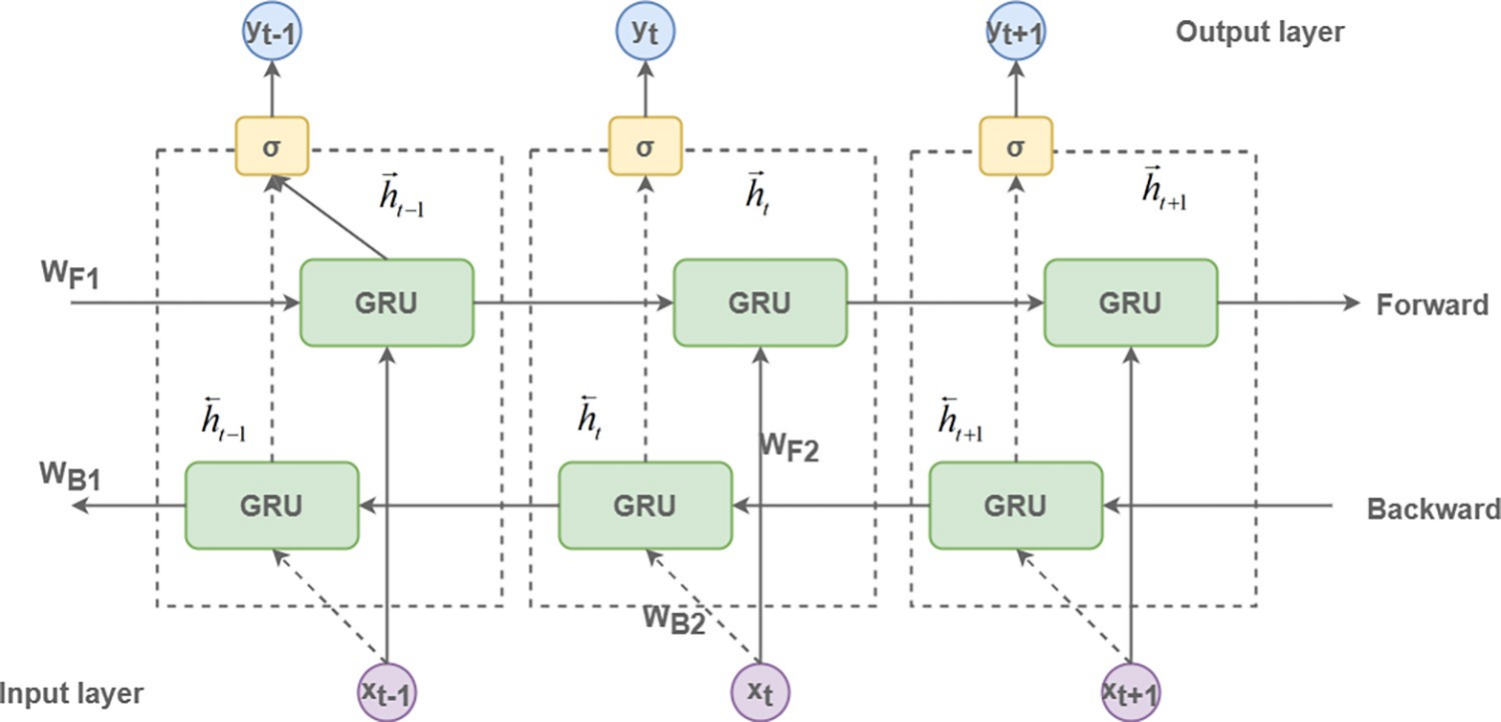
BiGRU framework diagram.

In the training of time-series data, the GRU employs unidirectional forward propagation, constraining its capability to fully elucidate the intricate relationships among target features. By contrast, the Bidirectional GRU (BiGRU) mitigates this constraint by utilizing both forward and reverse temporal propagations, thereby enabling an exhaustive examination of feature interdependencies. Specifically, the BiGRU architecture integrates a forward and a backward propagation layer; the former processes the sequence in a temporal manner, while the latter does so in reverse. This dual-layer configuration enhances the model’s effectiveness in predicting time-series data, as illustrated in Fig 9.

At time step t, the combined output from the BiGRU computational unit is given by:

$${\vec{h}}_{t}=\sigma (W_{F1}x_{t}+W_{F2}{\vec{h}}_{t-1}+b)$$ (12)

$${\overset{\leftarrow }{h}}_{t}=\sigma (W_{B1}x_{t}+W_{B2}{\overset{\leftarrow }{h}}_{t-1}+b)$$ (13)

$$y_{t}={\vec{h}}_{t}+{\overset{\leftarrow }{h}}_{t}$$ (14)

Here, $\vec{h_{t}}$ denotes the forward propagation, while $\overset{\leftarrow }{h_{t}}$ signifies the backward propagation. The vector associated with the output layer is represented by y_t_. Additionally, W serves as the weight matrix, and b corresponds to the bias vector.

### CNN-BiGRU model

**Fig 10:**
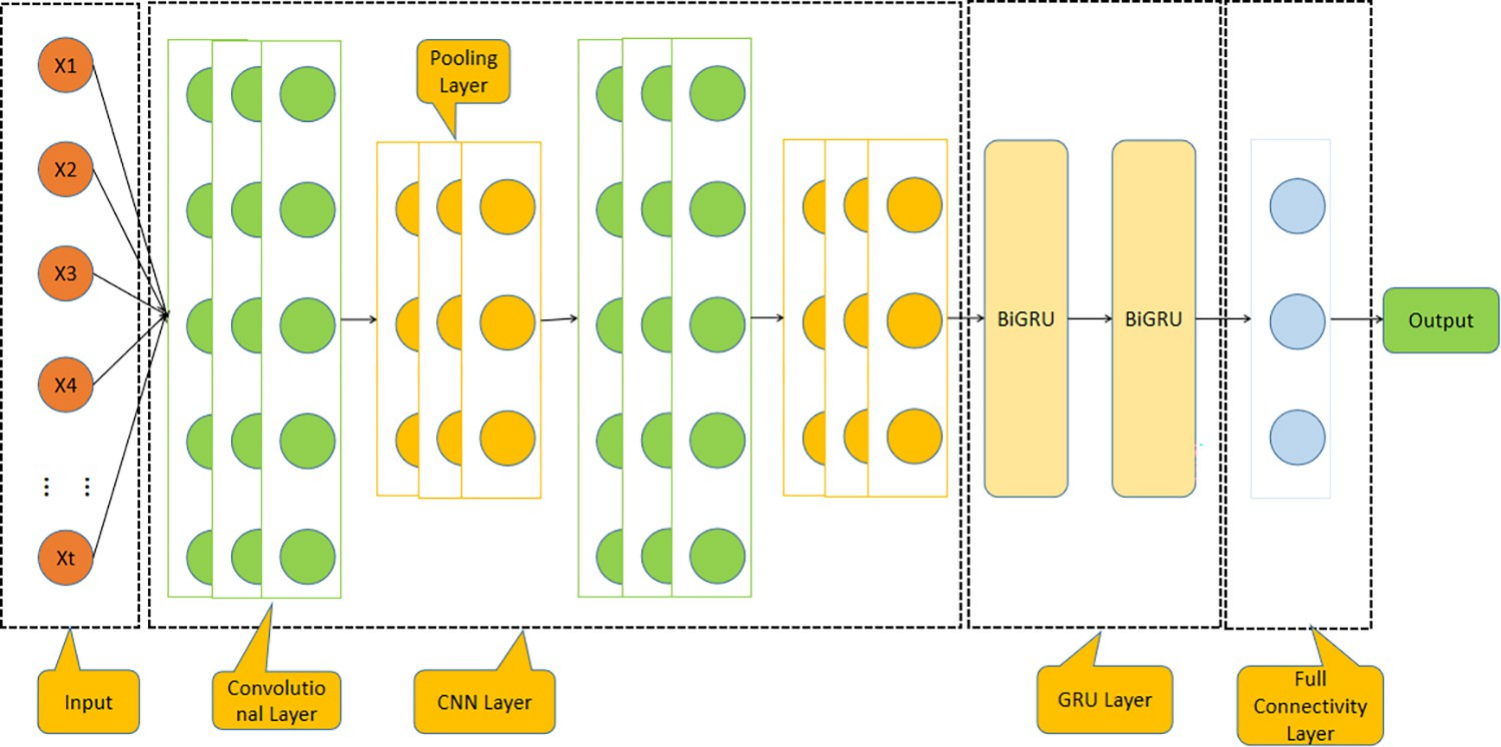
Hybrid modelling framework diagram.

In the present study, we introduce a hybrid model architecture, illustrated in Fig 10, designed for predictive analysis of hourly PM2.5 concentrations. The model ingests a dataset comprising eigenvalues obtained from the preceding 24-hour window as input variables. This framework marks a significant departure from our prior single-network strategy, enhancing prediction accuracy through a two-pronged computational approach.

The optimal values of the model we set according to the parameters of previous papers, so some of them are set according to the empirical values. The front-end of the model employs the CNN for sophisticated feature extraction. its parameters are delineated in Table 2. Following this, the extracted features are fed into the BiGRU module situated at the back-end, responsible for time-dependent PM2.5 concentration forecasting. The model is constructed as a two-layer deep neural network, its parameters are delineated in Table 3.

**Table 2:** CNN model parameter list.

Parameter names	The set value
kernel_size	3
MaxPooling	2
Num_layers	2
Activation Function	relu
DropOut	0.1
Stride	1
filters	24

**Table 3:** BiGRU Neural model parameter list.

Parameter names	The set value
Activation Function	selu
Loss Function	MSE+L2
Optimization algorithm	Adam
Epochs	200
Hidden_size	128
DropOut	0.1
Batch_size	128
Num_layers	2
Output_size	1
Input_size	24

To mitigate the risk of overfitting, we incorporate a dropout technique with a dropout rate of 0.1, effectively nullifying approximately 10% of the neurons in each hidden layer during the training phase. As for the activation function, the model employs Scaled Exponential Linear Units (SELU) owing to its superior convergence properties and effectiveness in addressing the vanishing gradient issue.

## Experiments

### Data

**Fig 11:**
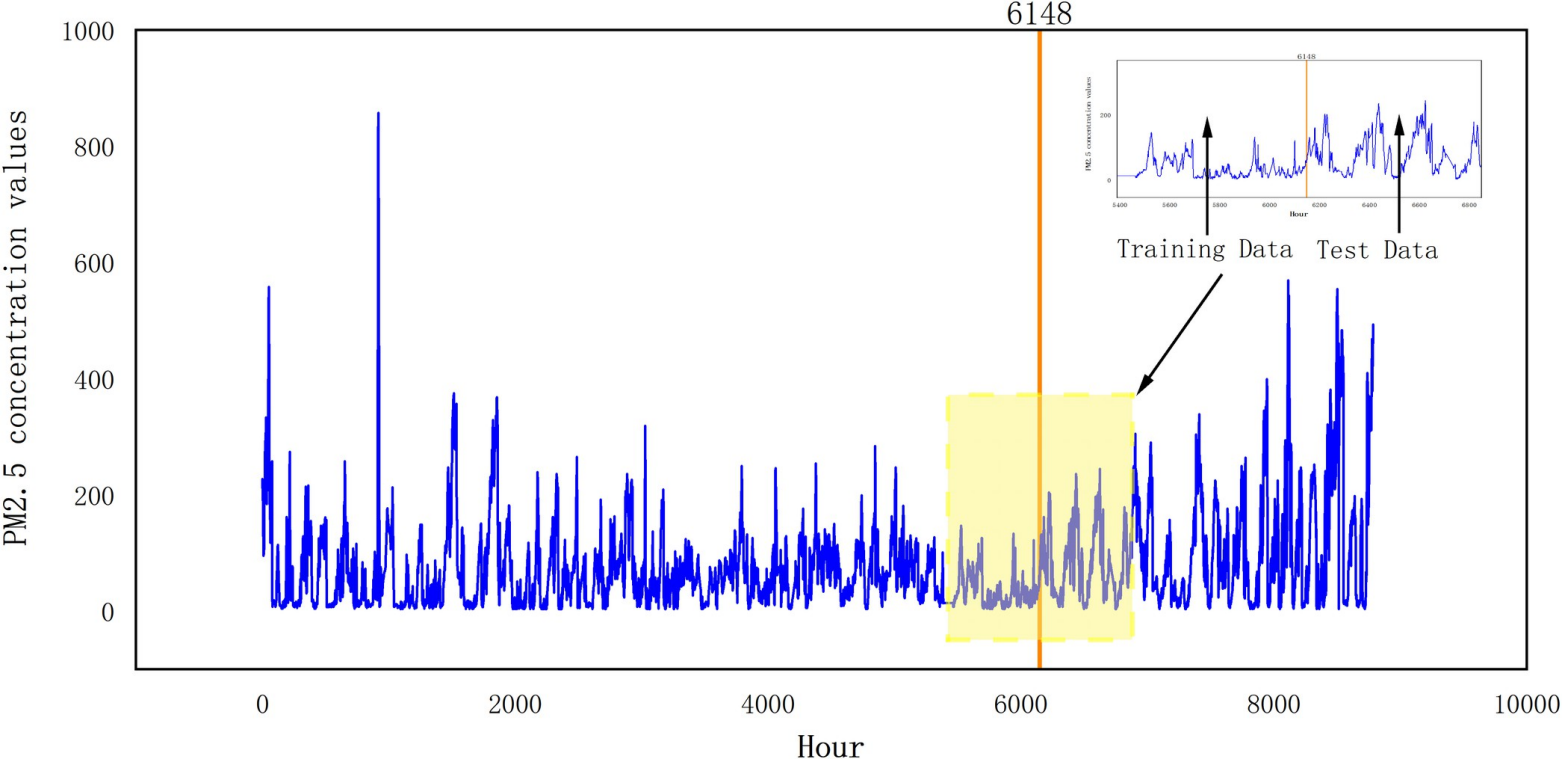
Map of the distribution of data sets.

CEEMDAN decomposition and seasonality and periodicity are related, the year is divided into spring, summer, autumn, and winter, and air quality is better in the seasons, such as spring and summer, CEEMDAN decomposition out of the time series will be relatively less, because the air pollutants are relatively more average, so the decomposition out of the IMF will be less; if it is the autumn and winter seasons, air quality is not good, the air quality value is more variable, then the IMF decomposed by CEMDAN will be relatively less. By the same token, time series with smoother periodicity will decompose less IMF, and time series with larger periodic fluctuation will decompose more IMF. So in this study, the data uses a full year’s worth of data, with both smooth and unsteady time periods. The data for this study was sourced from the Environmental Cloud of Nanjing YunChuang Big Data Technology Co., and includes hourly meteorological (AQI, air temperature, body temperature, barometric pressure, humidity, rainfall, wind speed) and air quality (PM2.5, PM10,…) data from Beijing, collected between January 1 and December 31, 2016. The dataset is time-series with 13 features related to air quality and meteorological factors, totaling 8784 instances at hourly intervals. For our experiments, we partitioned the data into two sets: 70% for training and 30% for testing, the following figure shows the distribution of data. Any missing data were imputed using linear interpolation.The exact distribution is shown in Fig 11.

### Evaluation indicators

This study employs three evaluation metrics: root mean square error (RMSE), mean absolute error (MAE), and R-squared (R^2^). RMSE quantifies the discrepancy between observed and true values, while MAE offers robustness against outliers. Lower RMSE and MAE values indicate less deviation in the model’s predictions. Conversely, a higher R^2^ value signifies a better fit of the predictive model. The equations for these metrics are provided as Eqs (15)–(17).


$$RMSE=\sqrt{\frac{1}{n}\sum_{i=1}^{n}{\left(y_{i}-{\bar{y}}_{i}\right)}^{2}}$$ (15)

$$MAE=\frac{1}{n}\sum_{i=1}^{n}\left|y_{i}-{\bar{y}}_{i}\right|$$ (16)

$$R^{2}=1-\frac{{\sum_{i=1}^{n}\left(y_{i}-{\hat{y}}_{i}\right)}^{2}}{\sum_{i=1}^{n}{\left(y_{i}-{\bar{y}}_{i}\right)}^{2}}$$ (17)

In this context, y_i_ represents the observed empirical value, ${\hat{y}}_{i}$ stands for the model’s predicted value, and ${\bar{y}}_{i}$ signifies the arithmetic mean of the observed empirical values.

### Ablation experiment

Ablation of neural networks is an experimental method to test the effect of certain parts of a neural network on the overall performance by gradually removing them. It can help to understand the functions and roles of different parts of a neural network and how much they contribute to the overall performance. Through ablation experiments, critical parts of a neural network can be identified to better understand how the neural network works and can provide guidance for improving the design and training of the neural network.

**Fig 12:**
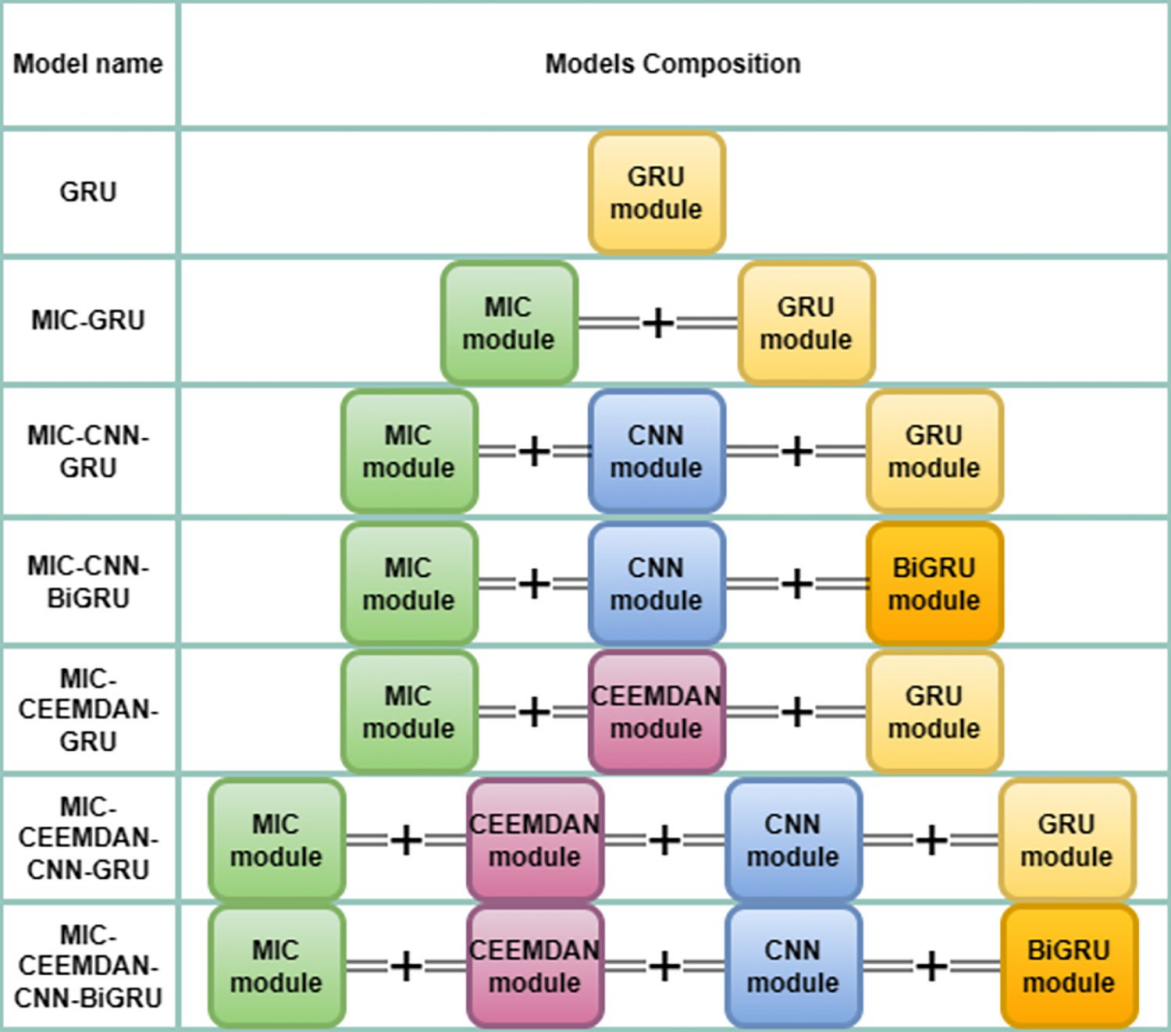
Comparison of ablation experimental models.

The ablation experiments in this study mainly focus on GRU, because GRU is easier to train than LSTM, and the training time of the GRU model is shorter, which is more efficient for short-time prediction performance, and avoids the shortcomings of the RNN model which is easy to gradient disappearance and gradient explosion. In this study, a total of seven models are designed for GRU ablation experiments, namely GRU, MIC-GRU, MIC-CNN-GRU, MIC-CNN-BIGRU, MIC-CEEMDAN-GRU, MIC-CEEMDAN-CNN-GRU and MIC-CEEMDAN-CNN-BiGRU proposed in this study. To ensure that the experiments are rational, the individual parameters of each model and module are the same. The comparison of each model for the ablation experiment is shown in Fig 12 below:

The calculated values of the error for each model of the ablation experiment can be seen in the next snippet Analysis of results, which contains not only the above seven models, but also a comparison of RNN and LSTM related models.

### Analysis of results

**Fig 13:**
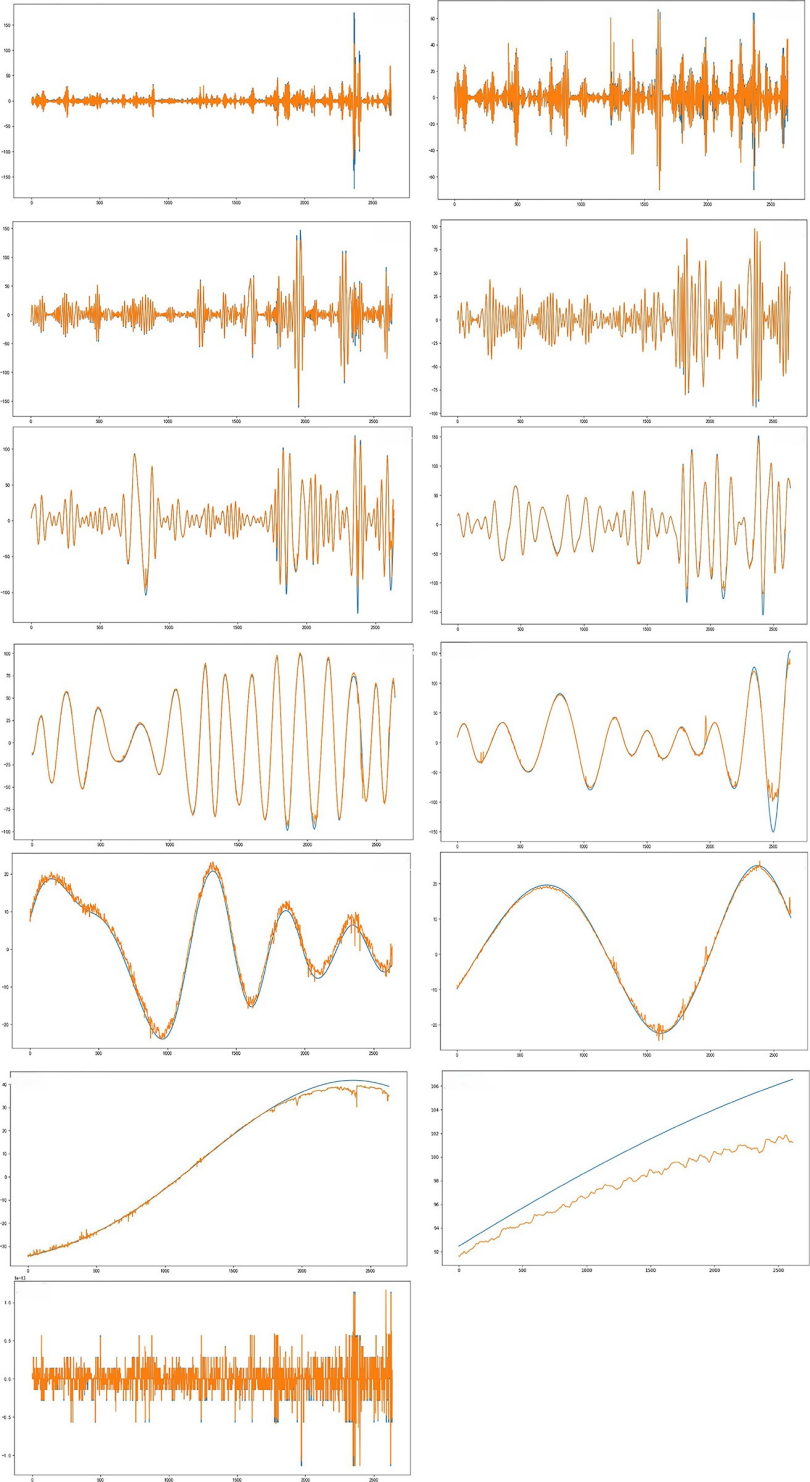
Decomposition prediction charts.

In this investigation, the final prediction results are derived from the aggregation of predicted values from each component, as outlined in Eq 18. Fig 13 presents the prediction results for each eigenmode function and residual, following the decomposition of the PM2.5 concentration time series using the MIC-CEEMDAN-CNN-BiGRU model. The actual PM2.5 values are represented in blue, while the predicted values from the proposed model are displayed in yellow. As can be seen from the lower Fig, there is a close alignment between the predicted and actual values across nearly all components.


$$y=\sum_{i=1}^{n}{\bar{C_{k}(t)}}^{'}+{r_{k}}^{'}(t)$$ (18)

In this formulation, y serves as the ultimate predictive value, n quantifies the number of instances of $\bar{C_{k}(t)}$ within the test set decomposition, and both ${\bar{C_{k}(t)}}^{\prime }$ and ${r_{k}}^{\prime }(t)$ constitute the predicted values specifically designated for the test set.

**Fig 14:**
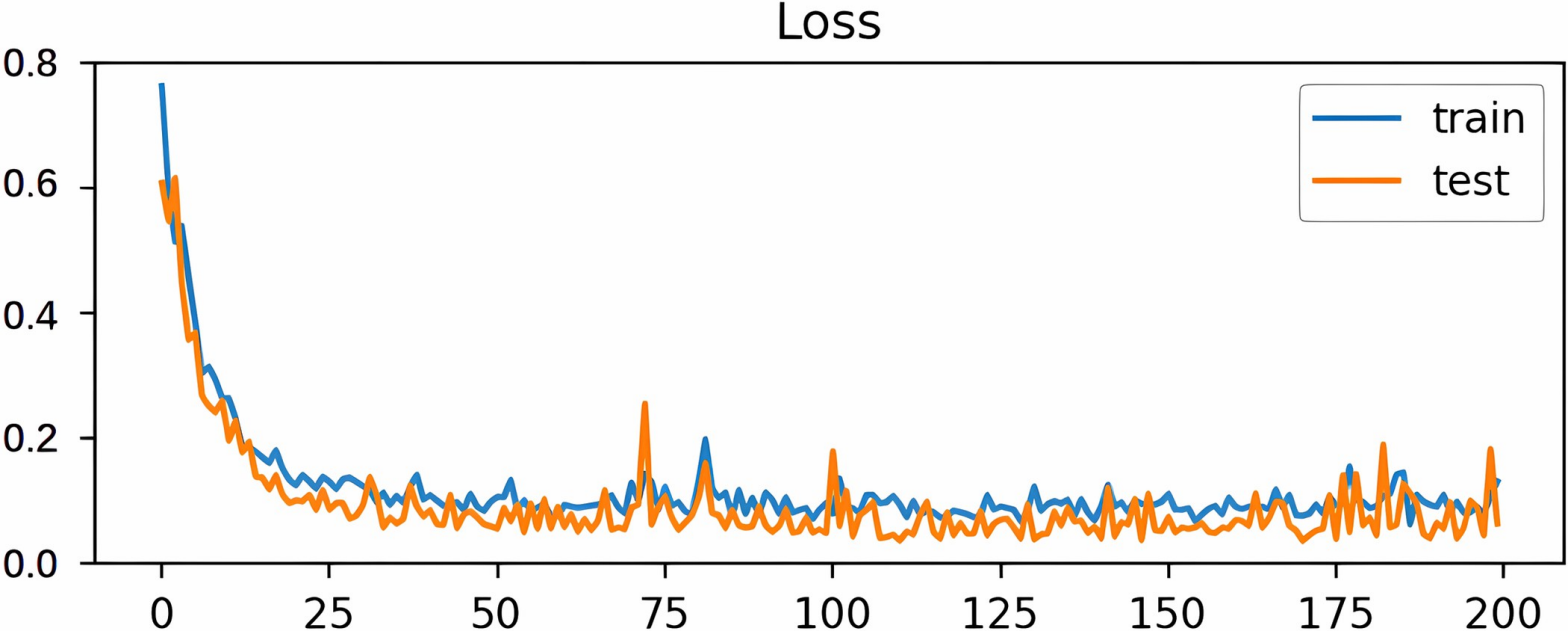
Training test loss plot.

**Fig 15:**
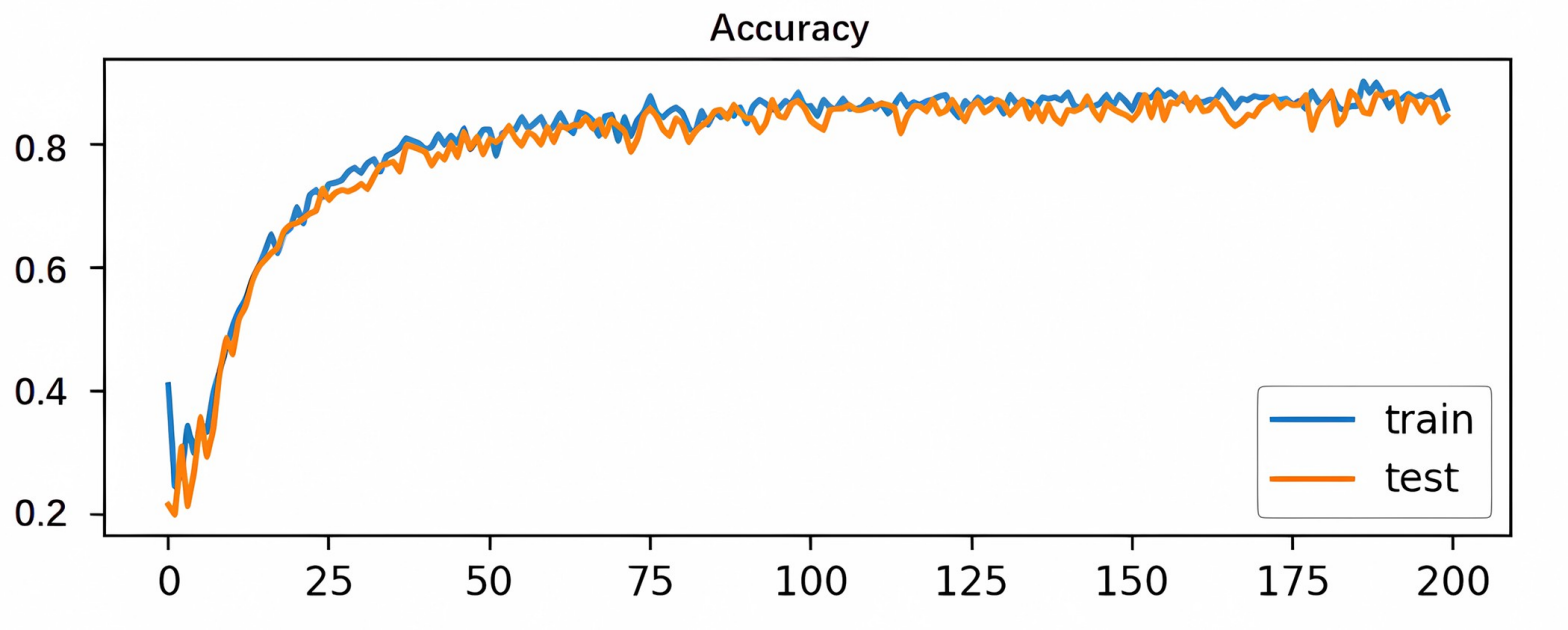
Training test accuracy plot.

The training process of a neural network model is designed to adjust the parameters of the model by learning the patterns and features of the input data to enable the model to accurately predict or classify new data. The training set accuracy measures how well the model performs on the training data, while the test set accuracy measures how well the model performs on never-before-seen data. Training set accuracy is used to assess how well the model fits the training data, while test set accuracy is used to assess the model’s generalization ability, how well the model can predict new data. Monitoring the training set and test set accuracies can help us understand how well the model is performing and can guide us in making adjustments and optimizations to the model. The training and testing process of the hybrid model in this paper is shown in Figs 14 and 15:

To validate the proposed method’s feasibility and accuracy, ten comparative prediction models were designed, encompassing RNN, MIC-RNN, LSTM, MIC-LSTM, GRU, MIC-GRU, MIC-CNN-GRU, MIC-CNN-BiGRU, MIC-CEEMDAN-GRU, and MIC-CEEMDAN-CNN-

GRU. All experiments were executed in a standardized environment. Model performance was assessed via three key metrics: MAE,RMSE, and R^2^, with findings summarized in Table 4.

**Table 4:** Evaluation index of the prediction results.

Models	Evaluation index		
	MAE	RMSE	R2
RNN	14.70	25.01	0.942
MIC-RNN	14.00	24.24	0.945
LSTM	12.75	26.22	0.935
MIC-LSTM	12.54	22.71	0.951
GRU	12.25	23.78	0.947
MIC-GRU	12.15	22.20	0.954
MIC-CNN-GRU	7.08	16.61	0.974
MIC-CNN-BIGRU	8.05	15.61	0.977
MIC-CEEMDAN-GRU	6.95	13.60	0.983
MIC-CEEMDAN-CNN-GRU	2.76	3.79	0.998
MIC-CEEMDAN-CNN-BiGRU	2.16	2.54	0.999

**Fig 16:**
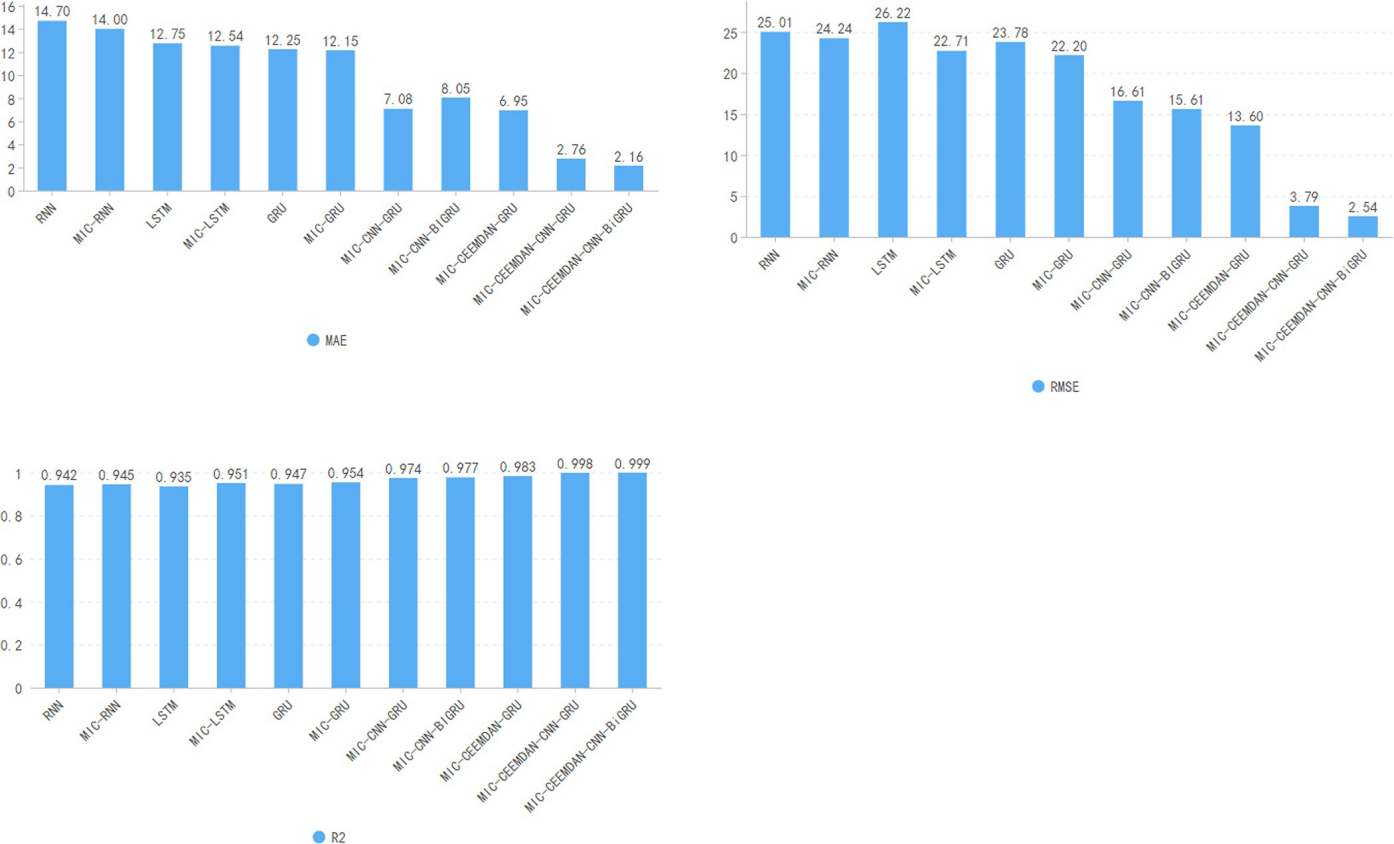
Indicators for the assessment of models.

**Fig 17:**
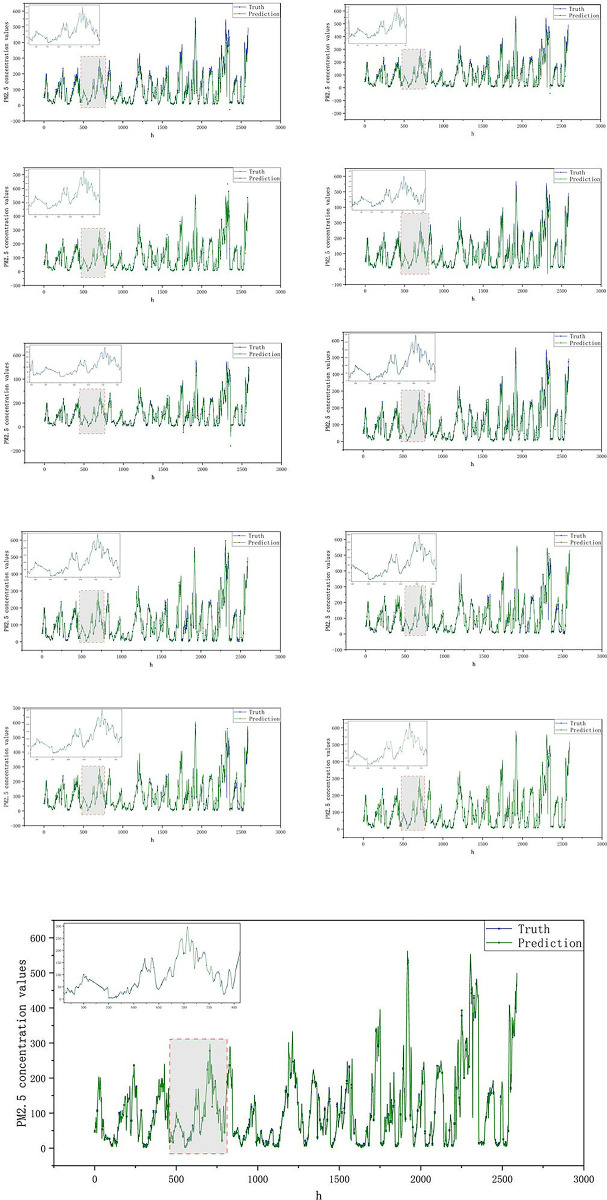
Comparison of model predictions.

It can be seen from Fig 16 that the results of the hybrid prediction model proposed in this paper are better than those predicted by the other models, based on the comparison of the values of the three indicators evaluated. The difference between the hybrid model of this paper and other hybrid models can be significantly seen in Fig 17. The plots in Fig 17 are arranged in order from left to right and from top to bottom to represent RNN, MIC-RNN, LSTM,MIC-LSTM,GRU,MIC-GRU,MIC-CNN-GRU,MIC-CNN-BIGRU,MIC-CEEMDAN-GRU, MIC-CEEMDAN-CNN-GRU, MIC-CEEMDAN-CNN-BiGRU.

## Conclusion

In this study, we rigorously examined the correlation between air quality factors, meteorological characteristics, and PM2.5 concentration, emphasizing the time series’ non-stationarity and its prediction implications. We introduced a novel model, MIC-CEEMDAN-CNN-BiGRU, which synergizes feature selection, data decomposition, and an integrated neural network, to predict short-term PM2.5 concentrations. Experimental evidence indicates that this hybrid model notably improves the accuracy of PM2.5 predictions, potentially serving as a robust tool for discerning concentration trends at specific monitoring sites, underscoring its practical value. The practical implications of PM2.5 prediction are manifold. For environmental protection, the prediction of PM2.5 concentration can help the environmental protection department monitor the air quality and take timely measures to control pollution and protect the environment; for public health, the prediction of PM2.5 can remind the public in advance to reduce outdoor activities and take protective measures to protect public health; for urban planning, the prediction of PM2.5 can help the urban planners to assess the impact of air quality on urban residents and guide urban planning. For urban planning, PM2.5 prediction can help urban planners assess the impact of air quality on urban residents, guide urban planning, and rationally plan industrial and residential areas, so as to reduce PM2.5 emissions and improve the urban environment.

However, this study’s model also presents constraints in its predictive capacity. Firstly, it focuses on PM2.5 concentration predictions at a single location within a distinct city, overlooking potential interconnections between multiple city-wide sites. For a holistic understanding, amalgamating data from all city monitoring sites is pivotal to unearth inter-site correlations. Such a comprehensive approach could yield more precise city-wide PM2.5 concentration predictions. Second, this research does not incorporate spatial characteristics of PM2.5, limiting the interpretation of model predictive variations across regions. Future endeavors should incorporate multimodal data to bolster prediction precision. Finally, the experimental results were not analysed qualitatively, which is easy to ignore and a weak issue for us and will be investigated as a later research direction.
